# Antibacterial and antifungal activities of natural deep eutectic solvents

**DOI:** 10.1007/s00253-024-13044-2

**Published:** 2024-02-07

**Authors:** Hadeer M. Bedair, Tamer M. Samir, Fotouh R. Mansour

**Affiliations:** 1 Department of Microbiology and Immunology, Faculty of Pharmacy, Misr University for Science and Technology (MUST), Giza, 12566 Egypt; 2 Pharmaceutical Analytical Chemistry Department, Faculty of Pharmacy, Tanta University, Elgeish Street, Tanta, 31111 Egypt

**Keywords:** Antibacterial, Antifungal, Natural deep eutectic solvents, Mechanism, Choline chloride

## Abstract

**Abstract:**

The increasing antibiotic resistance towards a panel of microorganisms is one of the public health concerns. For this reason, the search for alternatives to the widely used antibiotic has been undertaken. In the era of sustainable chemistry, deep eutectic solvents (DESs) have emerged as promising antimicrobial agents. These solvents possess several advantages such as low volatility, low flammability, ease of preparation, and typically low cost of production. These properties make DES suitable for various applications, including extraction of biomolecules and preparation of cosmetics. Natural DESs (NADESs) are special category of DESs prepared from natural sources, which matched the recent trends of leaning back to nature, and decreasing dependence on synthetic precursors. NADES can be prepared by heating and stirring, freeze-drying, evaporation, grinding, and ultrasound-assisted and microwave-assisted synthesis. Utilizing NADESs as an alternative to traditional antibiotics, which become ineffective over time due to bacterial resistance, holds great promise for these reasons. This review aims to discuss the antimicrobial properties of multiple NADESs, including antibacterial and antifungal activities. To the best of our knowledge, this review is the first literature survey of the antimicrobial activities of NADESs.

**Key points:**

• *Natural deep eutectic solvents are promising antimicrobial alternative to antibiotics*

• *NADES holds high potential for their activity against bacterial resistance*

• *NADES have also substantial antifungal activities*

## Introduction

Microbial infection is one of the greatest challenges facing the world, due to its serious clinical manifestations. Many researchers have been doing their best to overcome this problem in the last few decades (Silva et al. 2019). One of their interventions was the discovery of antibiotics, which was a turning point in medical therapies for combating microbial infections. However, each discovery was followed by the rise of antibiotic resistance, leading to the emergence of multidrug-resistant bacteria. The World Health Organization (WHO) has published a list of the most serious bacteria that are resistant to current treatments and require the development of new antibiotics to combat resistance.

Efforts have been made in research to meet the urgent demand for novel treatment approaches other than antibiotics. Biological alternatives to antibiotics against serious pathogens have been investigated. Antimicrobial proteins, bacteriophages, probiotics, and plant-based compounds are among the most promising alternatives (Łojewska and Sakowicz 2021). It has been known for centuries that plants possess natural antibacterial properties, in addition to their effectiveness against antibiotic-resistant microorganisms (Ng et al. 2021). They also contain volatile oil compounds (VOCs). These VOCs are molecules that are biosynthesized by primary and secondary metabolic pathways. VOCs include chemical classes such as alcohols, esters, aliphatic and aromatic hydrocarbons, terpenes, nitrogen, and sulfur compounds (Garrido et al. 2020). Additionally, the development of new green solvents that exhibit better properties than organic solvents is a key feature in green chemistry (Silva et al. 2019). Examples of these green solvents are room-temperature ionic liquids (RTILs) and deep eutectic solvents (DES). RTILs are very promising replacements for traditional volatile organic solvents as antibacterial treatments (Fernandes et al. 2022). They are composed of an organic cation and an inorganic or organic anion. They are attractive in research due to their versatility for an increasing number of technological applications. The main advantages of RTILs are high mobility, low melting points, negligible vapor pressure, thermal stability, low toxicity, large electrochemical window, and no flammability (Fernandes et al. 2022). However, experts frequently question their biodegradability and biological toxicity (Usmani et al. 2023).

On the other side, DESs have emerged as good alternatives to traditional organic solvents as well as their counterparts, ionic liquids (ILs) (Silva et al. 2019). DESs are mixtures of different ingredients that transition to liquid due to a massive depression in the melting point and charge delocalization generated by hydrogen bonding (Usmani et al. 2023). Moreover, another green solvent that has been developed is natural deep eutectic solvents (NADESs). NADESs are a new revolutionary class of green media that have now emerged as significant endeavor (Mouden et al. 2017), prepared from natural combinations of sugars, polyalcohols, sugar-based alcohols, amino acids, and organic acids. These liquid supermolecules are made up of economically viable and readily available constituents such as quaternary ammonium salts (e.g., choline chloride or betaine), hydrogen bond acceptors (HBA), and naturally derived uncharged hydrogen bond donors (HBD) (such as amines, sugars, acids, and alcohols). These solvents have numerous benefits, including easy synthesis, tunable physicochemical qualities, low toxicity, high biodegradability, solute sustainability and stabilization, and a low melting point (Usmani et al. 2023). NADESs have been widely used as media for chemical and enzymatic reactions, essential oil extraction, nanocarrier for drug delivery, and antioxidant and antimicrobial agents (Liu et al. 2018). In this mini-review, we will discuss the antibacterial, and the antifungal, activities of NADESs towards a panel of microorganisms. The novel and critical aspects of addressing the global challenge of microbial infections using these natural materials are elaborated. This review addresses a pressing need for innovative solutions in the fight against microbial infections. As far as we know, this review represents the first comprehensive survey of the antimicrobial potentials and activities of NADESs in the literature.

## NADESs preparation

**Fig. 1:**
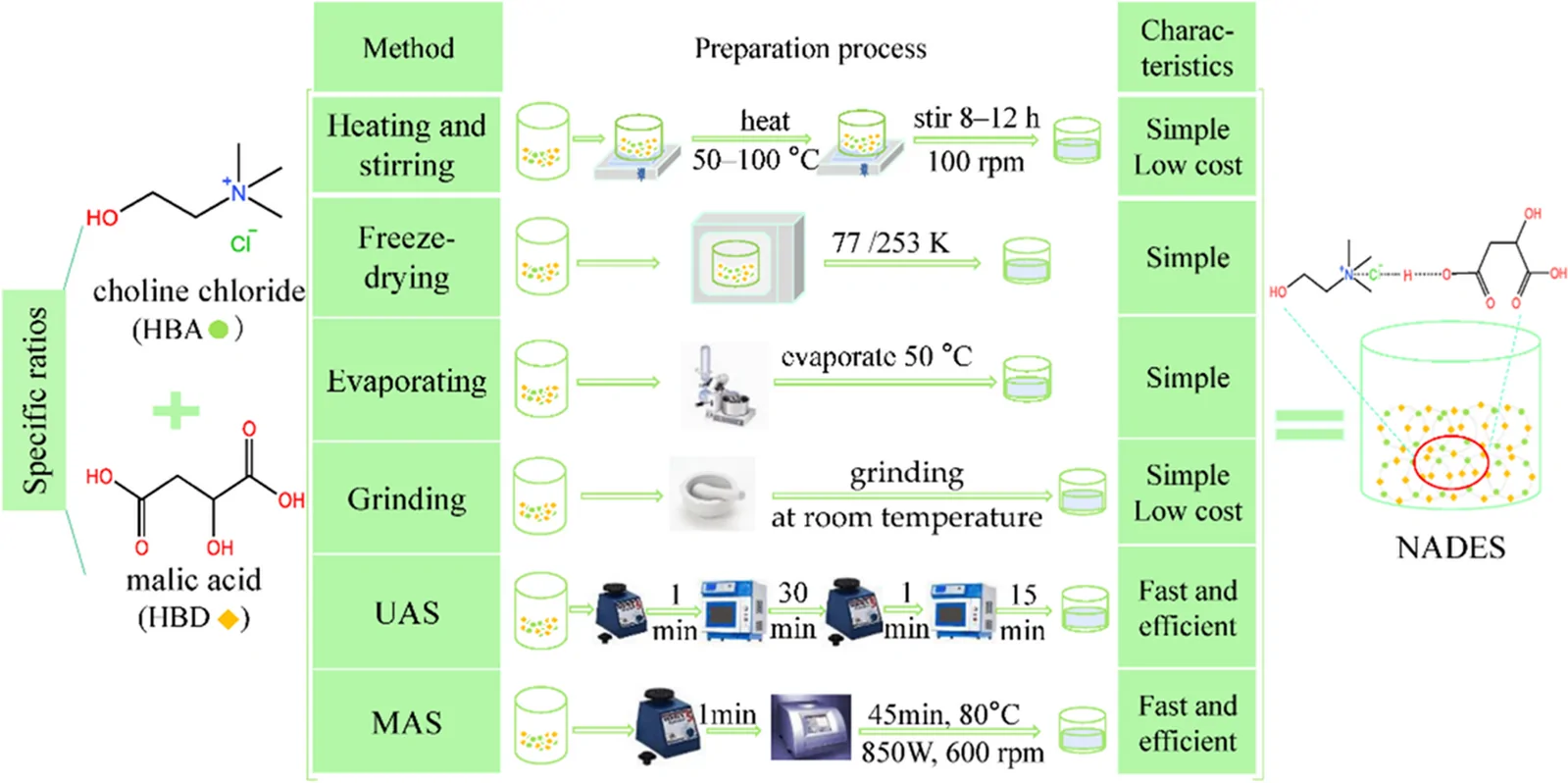
Different methods for the preparation of natural deep eutectic solvents from malic acid and choline chloride, with the permission of Wu et al. (2022)

NADES are typically prepared using one of six physical methods: heating and stirring, freeze-drying, evaporation, grinding, and ultrasound-assisted and microwave-assisted synthesis, as show in Fig. 1. Heating and stirring is the most commonly used method in NADESs preparation, due to its simplicity and low cost (Florindo et al. 2014; Castro et al. 2018). Another technique is vacuum evaporation, which involves heating a mixture of NADES components under reduced pressure to remove excess water and obtain a homogeneous liquid (Dai et al. 2013). Ultrasound-assisted synthesis utilizes sound waves to create cavitation, promoting the development of NADES (Rutkowska et al. 2017). Microwave-based methods subject the precursors to microwave energy, leading to molecular agitation and faster NADES formation (Gomez et al. 2018). These methods have been employed by different researchers, each with specific variations in the procedures and conditions. Overall, these diverse synthesis techniques contribute to the preparation and development of NADES. More information about these preparation methods can be find in recent reviews (Mišan et al. 2020; El Achkar et al. 2021; Omar and Sadeghi 2022; Wu et al. 2022).

## Techniques used to evaluate antimicrobial activity of NADESs

Different methods were used to evaluate the antimicrobial activity of NADESs against a broad category of microorganisms, including disc diffusion, well diffusion, broth dilution, Microtox assay, drop plate, and Fourier-transform infrared spectroscopy (FTIR) bioassay (Marchel et al. 2022). Among these methods, disc diffusion method was the most commonly used due to its ease of use (Marchel et al. 2022). In the next sections, we will briefly discuss each method.

### The disc diffusion method

The disc diffusion method, also known as the Kirby-Bauer test or the agar diffusion test, was first applied to determine the sensitivity of microorganisms to antibiotics (Jorgensen and Turnidge 2009). Recently, this method has been expanded to study the antimicrobial effect of some chemical compounds such as ILs (Ventura et al. 2012) and DES (Hayyan et al. 2013). Nowadays, it is being used to evaluate the antimicrobial toxicity of NADESs (Marchel et al. 2022).

In this test, a filter paper disc that has been impregnated with the testing substance is placed on the surface of an agar plate that has previously been uniformly swabbed with microorganisms. Then, the plate is put in an incubator with optimum growth conditions (temperature, time). During the incubation time, the microorganisms are allowed to grow, and the tested compound diffuses from the agar plate, inhibiting the growth of the microorganism. The result is obtained by measuring a clear zone of inhibition and comparing it with clinical laboratory standard institutes (Jorgensen and Turnidge 2009).

The main benefits of the disk diffusion method are that it is a cost-effective procedure, easy to perform, and easy to evaluate. On the other hand, the main disadvantage of this approach is that it only allows determining if the tested agent is toxic or non-toxic to the microorganism (Marchel et al. 2022). Therefore, it is recommended to determine the minimum inhibitory concentration (MIC) for the same pair of tested compound and microorganism in order to obtain accurate results.

The well diffusion method is another technique, similar to the disk diffusion method (Juneidi et al. 2015). In the well diffusion method, the liquid antimicrobial agents are added to wells in the agar plate, instead of using paper or plastic disks, as in the disks diffusion method. Accordingly, the concentration of the antimicrobial agent depends on the volume added to the wells, which affects the consistency of the results. This may explain why the disk diffusion method is much more commonly used than the well diffusion method. The results found in the literature for the antibacterial activity of NADESs against microorganisms using the disk diffusion method are summarized in Table 1.

**Table 1:** Applications of NADES as antibacterial agents

Bacteria	Strain	Inhibition zone(mm) ± SD	MIC	HBD	HBA	Ratio (HBD/HBA)	Preparation method	Preparation time (min)	Ref
*Arthrobacter simplex*	TCCC 11037	19.8 ± 4.1	NR	glycerol	Choline chloride	2:1	Thermal mixing	60–120	Mao et al. (2018)
*Bacillus subtilis*	ATCC 6633	50 ± 5 mm	0.012 ± 0.002 µL/mL	Acetic acid	Choline chloride	2:1	Heating and stirring	NR	Mouffok et al. (2023)
*Clavibacter michiganensis* subsp. *michiganensis*	CECT 790	NR	150 × 10^3^ mg/L	Sucrose	Betaine	1:2	Heating and stirring	15–30	Rodríguez-Juan et al. (2021)
*Clavibacter michiganensis* subsp.* insidious*	CECT 5042	NR	38 × 10^3^ mg/L	Sucrose	Betaine	1:2	Heating and stirring	15–30	Rodríguez-Juan et al. (2021)
*Clostridium perfringens*	ATCC 13124	(13–17) IE = 40.61b%	NR	1,2-Propanediol	Choline chloride	2:1	Heating and stirring	150	Wojeicchowski et al. (2021)
*Escherichia coli*	NR	25.50 ± 0.5	NR	Lactic acid	Menthol	2:1	Heating and stirring	NR	Alsaud et al. (2021)
*Enterococcus faecalis*	ATCC 19433	50 ± 5 mm	0.20 ± 0.1 µL/mL	Acetic acid	Choline chloride	2:1	Heating and stirring	NR	Mouffok et al. (2023)
*Erwinia toletana*	CECT 5263	NR	300 × 10^3^ mg/L	Sucrose	Betaine	1:2	Heating and stirring	15–30	Rodríguez-Juan et al. (2021)
*Escherichia coli*	ATCC 25922	42 ± 3 mm	0.05 ± 0.01 µL/mL	Acetic acid	Choline chloride	2:1	Heating and stirring	NR	Mouffok et al. (2023)
*Escherichia coli*	NR	24.8 ± 0.3	12 mmol/L	Oxalic acid	Choline chloride	1:1	Heating and stirring	120–240	Zhao et al. (2015)
*Escherichia coli*	K12 DSM498	NR	5 mg/mL	Mandelic acid	Choline chloride	2:1	Heating and stirring	NR	Mano et al. (2017)
*Escherichia coli*	ATCC 8739	NR	500 µg/mL	Decanoic acid	Menthol	1:2	Heating and stirring	60	Syed et al. (2020)
*Escherichia coli*	3014	47 ± 2	NR	Oxalic acid	Choline chloride:	1:1	Heating and stirring	120–360	Radošević et al. (2018)
*Escherichia coli*	3014	50 ± 4	NR	Fructose/glycerol	Citric acid	1:1:1	Heating and stirring	120–360	Radošević et al. (2018)
*Escherichia coli*	ATCC-23564	29	NR	Oxalic acid/EG	Choline chloride	1:1:1	Heating and stirring	NR	Jangir et al. (2020)
*Escherichia coli*	NR	7.12	NR	Malic acid	Betaine	1:1	Ultrasonic irradiation	30	Liang et al. (2020)
*Escherichia coli*	NR	Inhibition index = 80%	NR	Urea	Choline chloride	1:1	Heating and stirring	60–120	Wen et al. (2015)
*Klebsiella pneumoniae*	Clinical isolate	50 ± 5 mm	0.10 ± 0.05 µL/mL	Acetic acid	Choline chloride	2:1	Heating and stirring	NR	Mouffok et al. (2023)
*Listeria monocytogenes*	NR	15 ± 0.1	14 mmol/L	Oxalic acid	Choline chloride	1:1	Heating and stirring	120–240	Zhao et al. (2015)
*Listeria monocytogenes*	ATCC 7644	(13–17) IE = 51.22a%	NR	1,2-Propanediol	Choline chloride	2:1	Heating and stirring	150	Wojeicchowski et al. (2021)
*Listeria. Monocytogenes*	Clinical isolate	40 ± 2 mm	0.10 ± 0.1 µL/mL	Acetic acid	Choline chloride	2:1	Heating and stirring	NR	Mouffok et al. (2023)
*MRSA*	ATCC 700698	16.50 ± 0.41	625 µg/mL	Lauric acid	Capric acid	1:2	Heating and stirring	30	Silva et al. (2019)
*MRSA*	ATCC 700,698	16.70 ± 0.47	1250 µg/mL	Lauric acid	Menthol	1:4	Heating and stirring	(5–10)	Oliveira et al. (2023)
*MRSE*	ATCC 35,984	12	1250 µg/mL	Lauric acid	Menthol	1:4	Heating and stirring	(5–10)	Oliveira et al. (2023)
*MRSE*	ATCC 35984	20.00 ± 0.82	625 µg/mL	Lauric acid	Capric acid	1:2	Heating and stirring	30	Silva et al. (2019)
*P. aeruginosa*	ATCC 9027	NR	20% of NADES	Glycerol	Choline chloride:	1:1	The vacuum evaporation	20	Nystedt et al. (2023)
*P. aeruginosa*	NR	28.50 ± 0.5	NR	Lactic acid	Menthol	2:1	Heating and stirring	NR	Alsaud et al. (2021)
*P. aeruginosa*	ATCC 27853	40 ± 5 mm	0.01 ± 0.005 µL/mL	Acetic acid	Choline chloride	2:1	Heating and stirring	NR	Mouffok et al. (2023)
*P. aeruginosa*	3024	50 ± 2	NR	Oxalic acid	Choline chloride:	1:1	Heating and stirring	120–360	Radošević et al. (2018)
*P. aeruginosa*	3024	51 ± 4	NR	Fructose/glycerol	Citric acid	1:1:1	Heating and stirring	120–360	Radošević et al. (2018)
*P. savastanoi*	CECT 5019	NR	150 × 10^3^ mg/L	Sucrose	Betaine	1:2	Heating and stirring	15–30	Rodríguez-Juan et al. (2021)
*P. syringae*	CECT 4429	NR	150 × 10^3^ mg/L	Sucrose	Betaine	1:2	Heating and stirring	15–30	Rodríguez-Juan et al. (2021)
*Proteus mirabilis*	3008	49 ± 1	NR	Oxalic acid	Choline chloride:	1:1	Heating and stirring	120–360	Radošević et al. (2018)
*Proteus mirabilis*	3008	81 ± 2	NR	Fructose/glycerol	Citric acid	1:1:1	Heating and stirring	120–360	Radošević et al. (2018)
*Proteus. mirabilis*	Clinical isolate	50 ± 5 mm	0.002 ± 0.001 µL/mL	Acetic acid	Choline chloride	2:1	Heating and stirring	NR	Mouffok et al. (2023)
*Rhizobium radiobacter*	CECT 4119	NR	150 × 10^3^ mg/L	Sucrose	Betaine	1:2	Heating and stirring	15–30	Rodríguez-Juan et al. (2021)
*Staphylococcus aureus*	ATCC 6538	NR	15.63 µg/mL	Decanoic acid	Menthol	1:2	Heating and stirring	60	Syed et al. (2020)
*Staphylococcus aureus*	ATCC 6538	NR	20–25% of NADES	Glycerol	Choline chloride:	1:1	The vacuum evaporation	20	Nystedt et al. (2023)
*S. epidermidis*	NR	33.00 ± 2	NR	Lactic acid	Menthol	1:2	Heating and stirring	NR	Alsaud et al. (2021)
*Salmonella enteritidis*	NR	19.3 ± 0.7	12 mmol/L	Oxalic acid	Choline chloride	1:1	Heating and stirring	120–240	Zhao et al. (2015)
*Salmonella* spp.	ATCC 13076	18 IE = 43.54b%	NR	1,2-Propanediol	Choline chloride	2:1	Heating and stirring	150	Wojeicchowski et al. (2021)
*Salmonella typhimurium*	3064	45 ± 4	NR	Oxalic acid	Choline chloride:	1:1	Heating and stirring	120–360	Radošević et al. (2018)
*Salmonella typhimurium*	3064	55 ± 1	NR	Fructose/glycerol	Citric acid	1:1:1	Heating and stirring	120–360	Radošević et al. (2018)
*Salmonella. typhimurium*	ATCC 14028	43 ± 3 mm	0.01 ± 0.007 µL/mL	Acetic acid	Menthol	1:1	Heating and stirring	NR	Mouffok et al. (2023)
*Serratia marcescens*	Clinical isolate	45 ± 5 mm	0.001 ± 0.005 µL/mL	Acetic acid	Choline chloride	2:1	Heating and stirring	NR	Mouffok et al. (2023)
*Staphylococcus aureus*	Clinical isolate	50 ± 5 mm	0.01 ± 0.002 µL/mL	Acetic acid	Choline chloride	2:1	Heating and stirring	NR	Mouffok et al. (2023)
*Staphylococcus aureus*	ATCC 25923	(13–17) IE = 47.92a%	NR	1,2-Propanediol	Choline chloride	2:1	Heating and stirring	150	Wojeicchowski et al. (2021)
*Staphylococcus aureus*	ATCC6538	26 ± 0.7	0.2 µg/mL	Capric acid	Menthol	1:1	Heating and stirring	1	Al-Akayleh et al. (2022)
*Staphylococcus aureus*	NR	19.7 ± 0.7	12 mmol/L	Oxalic acid	Choline chloride	1:1	Heating and stirring	120–240	Zhao et al. (2015)
*Staphylococcus aureus*	NCTC8325	NR	5 mg/mL	Mandelic acid	choline chloride	2:1	Heating and stirring	NR	Mano et al. (2017)
*Staphylococcus aureus*	*ATCC 25,923*	14.17 ± 0.62	625 µg/mL	Lauric acid	Menthol	1:4	Heating and stirring	(5–10)	Oliveira et al. (2023)
*Staphylococcus aureus*	3048	73 ± 3	NR	Oxalic acid	Choline chloride:	1:1	Heating and stirring	120–360	Radošević et al. (2018)
*Staphylococcus aureus*	3048	51 ± 3	NR	Fructose/glycerol	Citric acid	1:1:1	Heating and stirring	120–360	Radošević et al. (2018)
*Staphylococcus aureus*	ATCC-9144	25	NR	Oxalic acid/EG	Choline chloride	1:1:1	Heating and stirring	NR	Jangir et al. (2020)
*Staphylococcus aureus*	ATCC 25923	15.67 ± 0.58	625 µg/mL	Lauric acid	Capric acid	1:2	Heating and stirring	30	Silva et al. (2019)
*Xanthomonas campestris*	CECT 97	NR	75 × 10^3^ mg/L	Sucrose	Betaine	1:2	Heating and stirring	15–30	Rodríguez-Juan et al. (2021)

*NR* Not reported

### Agar and broth dilution technique

It has been reported that agar or broth dilution methods are among the most commonly used techniques to evaluate the antimicrobial activity of NADESs (Marchel et al. 2022). These methods allow for the determination of MIC of the studied antimicrobial agent.

In the agar dilution technique, a predetermined inoculum of microorganisms is directly applied onto nutrient agar plates containing different concentrations of the tested antimicrobial agent (Wiegand et al. 2008). The plates are then incubated under optimum conditions (e.g., time, temperature) for the growth of the tested microorganism. After the incubation period, the results are observed. Colonies on the plates indicate the growth of the microorganism, while the plate with the lowest concentration of the studied molecule where the bacteria did not grow specifies its MIC value (Wiegand et al. 2008).

The main advantage of the agar dilution method is that it is suitable for evaluating a large number of bacterial isolates against a small number of antimicrobial agents with limited concentrations (Kadlec et al. 2015). However, the main disadvantages of this method are that it is time-consuming and requires a large number of plates with varying concentrations of the antimicrobial agent (Kadlec et al. 2015). This makes it cost-ineffective due to the requirement of a substantial amount of the tested antimicrobial agent with different concentrations (Kadlec et al. 2015).

In the broth dilution method, the bacteria are grown in liquid nutrient medium containing the antibiotic in increasing concentrations (usually in twofold dilution series). A certain number of microbial cells are then inoculated into the medium. The main advantages of the broth dilution method are its ease of application, the ability to test the sensitivity of microorganisms to several chemicals simultaneously (Wiegand et al. 2008), and obtaining quantitative results. However, the disadvantages of this method are similar to those of the agar dilution method (Kadlec et al. 2015).

### Microtox assay

**Fig. 2:**
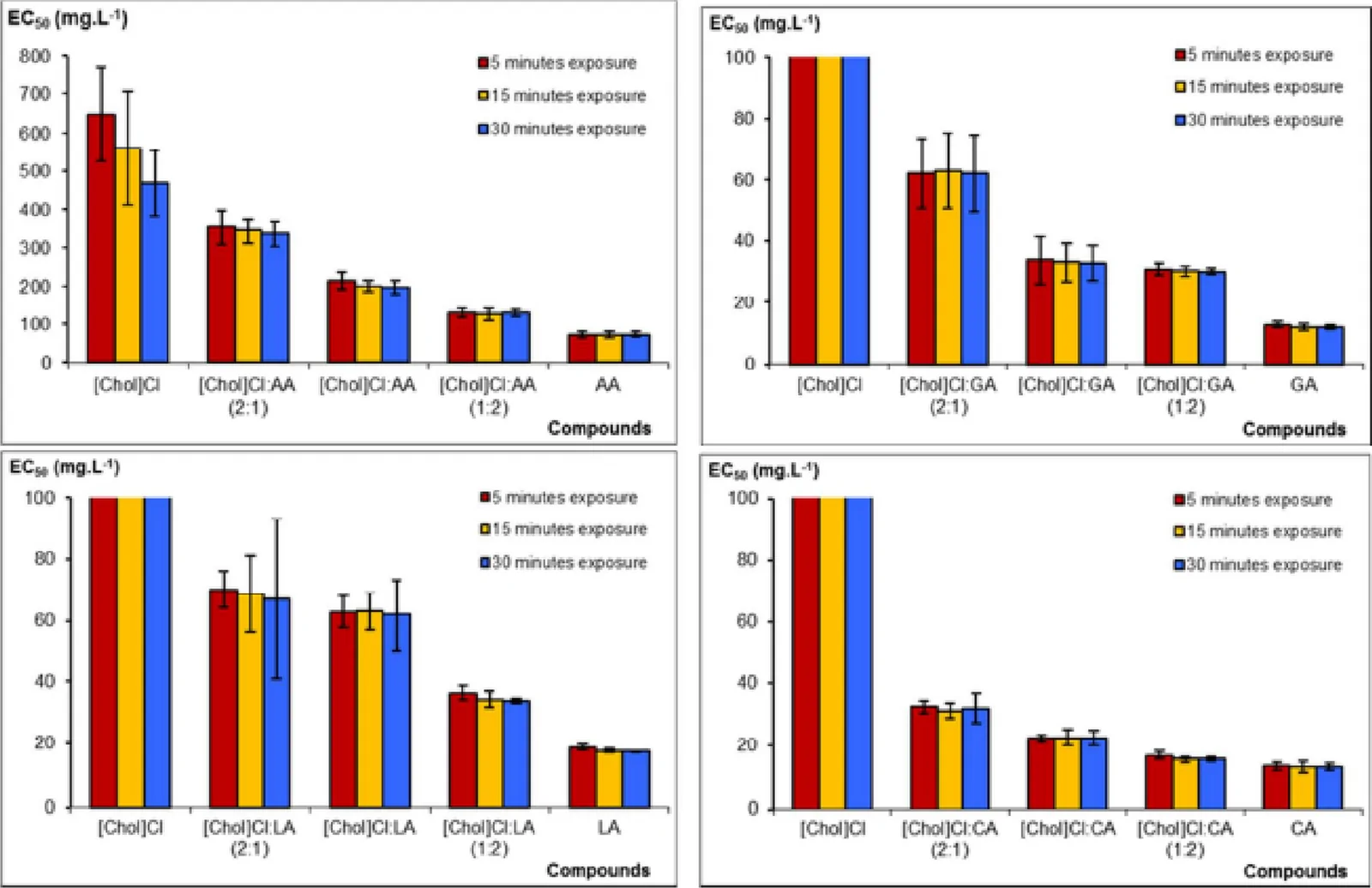
Values of median EC50, in mg L^−1^, obtained after 5, 15, and 30 min of exposure of the marine bacteria *Vibrio fischeri* (Microtox.® toxicity test) to different chemical compounds, with the permission from De Morais et al. (2015)

In this assay, the toxicity of different substances can be determined by using bioluminescent bacteria *Aliivibrio fischeri*, which are non-pathogenic marine microorganisms that naturally luminesce as part of their metabolism (Johnson 2005). The toxic agent disrupts the respiratory process of these bacteria, resulting in a decrease in light output (Johnson 2005). The main advantages of this assay are its rapidity, ease of use, and sensitivity. In addition, a strain of bioluminescent bacteria is available in a lyophilized vial format, which increases their shelf life and usability (Johnson 2005). The in vitro toxicity of the NADESs was evaluated using the Microtox assay, for the first time, in the work of De Morais et al. (2015) who used cholinium chloride as hydrogen bond acceptor and organic acids as hydrogen bond donor. The results indicated that the four carboxylic acids (acetic, citric, lactic, and glycolic acids) presented a moderate toxicity (De Morais et al. 2015), in the following ascending order: acetic acid < lactic acid < citric acid < glycolic acid. It was observed that the effective concentration (EC50), in mg/L, obtained after 5 min was 30.9 for choline chloride: glycolic acid at molar ratio of 1:2 (De Morais et al. 2015). However, the level of toxicity of these different acids towards *Aliivibrio fischeri* was similar, except for acetic acid, being 4 to 5 times less than the other acids. Figure 2 illustrates the values of EC50, after different durations of exposure of the marine bacteria *Vibrio fischeri* to different NADES. Although organic acids resulted in lower EC50, and hence more antimicrobial efficiency, NADES are usually more potent than its starting compounds. These results show how Microtox assay can be beneficial to compare between newly prepared NADES.

### Drop plate method

**Fig. 3:**
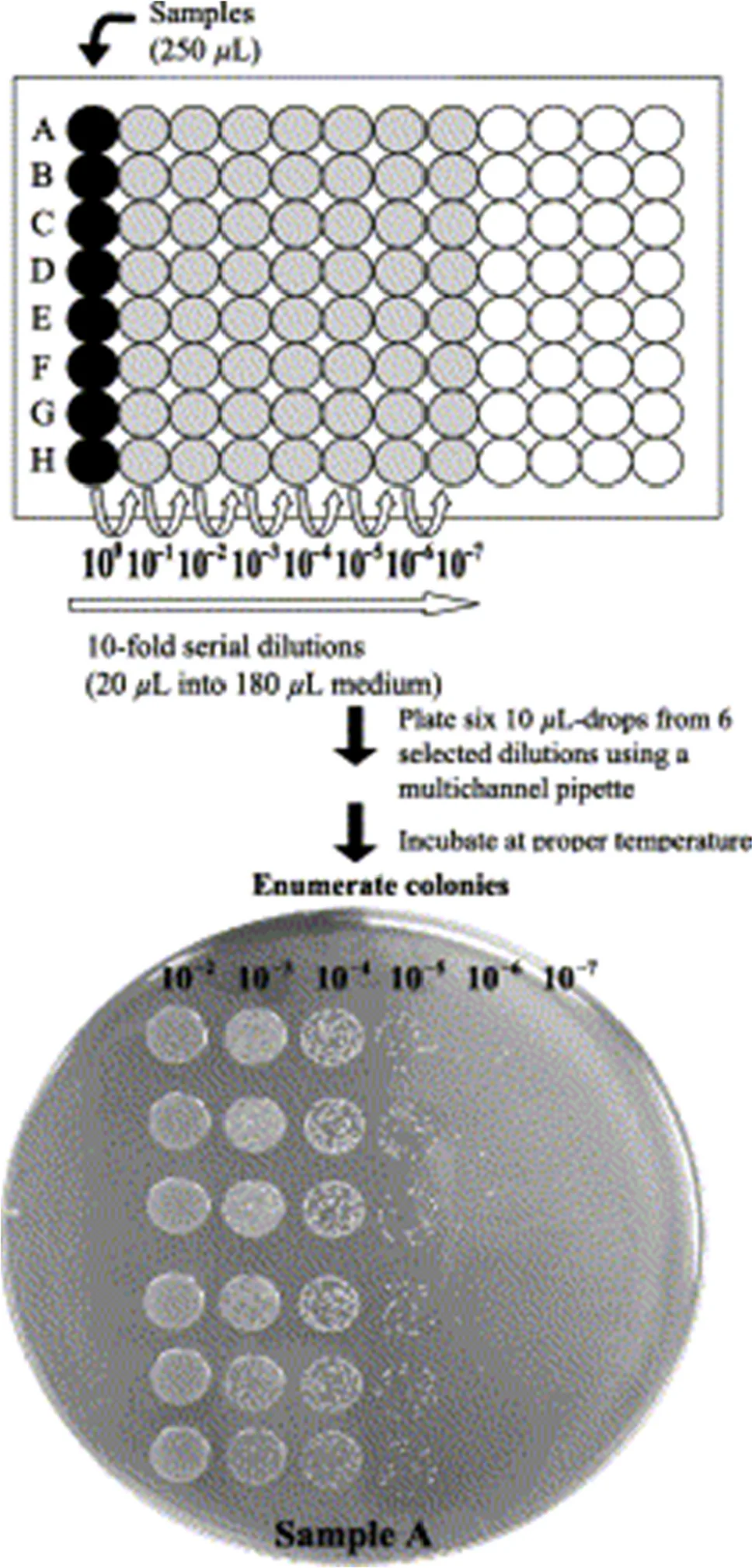
Results of the drop plate method for studying *Campylobacter jejuni* (1 to 10 serial dilutions). With the permission of Chen et al. (2003)

In this method, a 24-well plates for serial dilutions are used, followed by drop plating on agar in a 4 × 4 format using an automatic spiral plater (Chen et al. 2003; Wikene et al. 2017). After that, the plates are left to dry for a few minutes and then placed into an incubator for 18–20 h (37 °C) (Chen et al. 2003; Wikene et al. 2017). After incubation, viable colony forming units (CFUs) are counted and compared to control samples. Figure 3 illustrates the schematic of the 6 × 6 drop plate method. The bottom photograph depicts 6 drops × six 1 to 10 serial dilutions of *Campylobacter jejuni*. In the study conducted by Wikene and his coworkers, the drop plate method was used to assess the toxicity of NADESs. The tested NADESs included citric acid/sucrose and glucose/malic acid, while the model microorganisms included *Escherichia coli* and *Enterococcus faecalis*. Notably, the results unveiled that the 100-fold dilutions of the aforementioned NADESs exhibited minimal toxicity towards the bacterial strains, as evidenced by the absence of a significant reduction in colony-forming units (CFUs) relative to the untreated control samples (Wikene et al. 2015a). Additionally, this method was employed to evaluate the toxic effect of glucose/sucrose and choline chloride/maleic acid NADESs on *Escherichia coli* (Wikene et al. 2015b). The results revealed that the carbohydrate-based NADESs were non-toxic to *Escherichia coli*, with no significant reduction in the viable bacterial count (Wikene et al. 2015b). In addition, the antimicrobial properties of choline chloride/xylitol, malic acid/fructose/glucose, and citric acid/sucrose NADESs against three strains of bacteria were explored using the drop plate method [35]. The results showed that citric acid/sucrose NADES was non-toxic to all three bacterial strains (*Escherichia coli*, *Enterococcus faecalis*, and *Staphylococcus aureus*) (Wikene et al. 2016), which supported the findings of Wikene et al. (2015a).

### FTIR-based biological assay

**Fig. 4:**
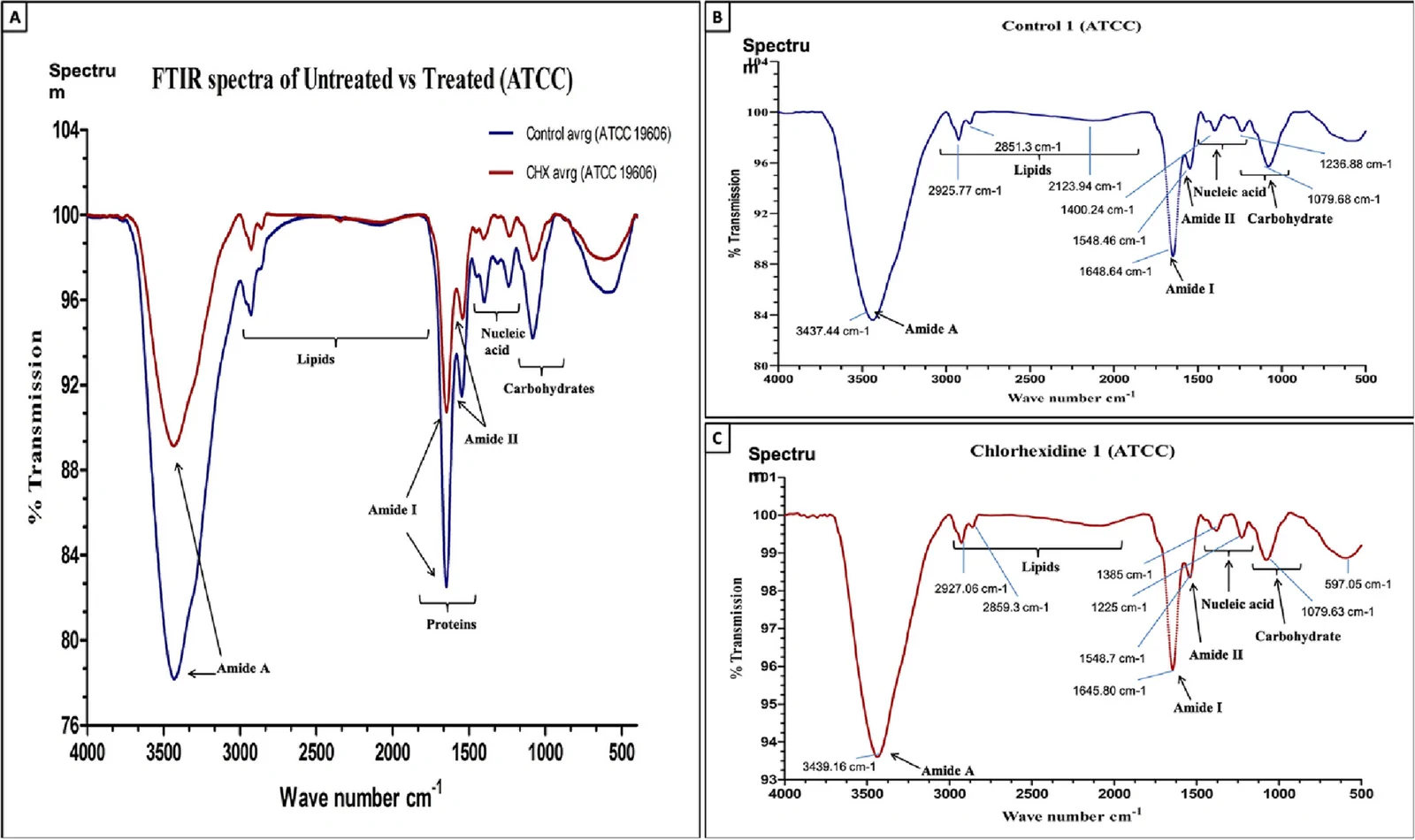
**A** FTIR spectra of *A. baumannii* (ATCC) before (blue) and after treatment with 32 µg/mL of chlorhexidine (CHX, red), **B** before treatment, and **C** after treatment with 32 µg/mL CHX. The experiment was performed in triplicates. With the permission of Biswas et al. (2019)

Another method used to evaluate the antimicrobial activity of NADESs depends on using FTIR spectroscopy, which is an instrumental technique used to elucidate the chemical composition of a substance by measuring the absorption, emission, or reflection of IR radiation. This technique is based on the principle that different chemical bonds vibrate at specific frequencies when exposed to IR. Thus, FTIR spectroscopy can be utilized to assess any changes in the molecular structure of the microorganisms or the NADESs themselves after exposure. FTIR is also capable of detecting changes in cell metabolites (Corte et al. 2010). This allows for a metabolomics analysis to be obtained. Since cells under stress exhibit rapid changes in their metabolites, this bioassay can estimate the toxicity level by measuring the variation in FTIR spectra of the cells upon exposure to the chemicals. Additionally, it provides metabolic indexes that can be used to classify and quantify the toxicity of the tested agent. The yeast strain of *Saccharomyces cerevisiae* cells has been commonly used as biosensor cells in this method. The main advantages of this approach are its speed and reproducibility (Corte et al. 2010). FTIR spectra are usually acquired using the potassium bromide (KBr) disc method in transmission mode in the range of 4000–400 cm^−1^. Sample pellets containing KBr were prepared by grinding in a mortar and pestle (Biswas et al. 2019). Background spectra from the KBr disc were subtracted from the spectra of the KBr and sample discs. The FTIR spectra of the untreated strains exhibit characteristic bands corresponding to biomolecules: lipids (3000–2800 cm^−1^), proteins/amides I and II (1700–1500 cm^−1^), phospholipids/DNA/RNA (1500–1185 cm^−1^), polysaccharides (1185–900 cm^−1^), and the fingerprint region (900–600 cm^−1^). The spectral analysis should reveal significant differences between untreated and treated samples, particularly in the absorbance of macromolecules such as proteins, nucleic acids, carbohydrates, and phospholipids, as shown in Fig. 4. These changes help elucidate the mechanism of action of the studied antimicrobial agent (Biswas et al. 2019).

### Electron microscopy techniques

**Fig. 5:**
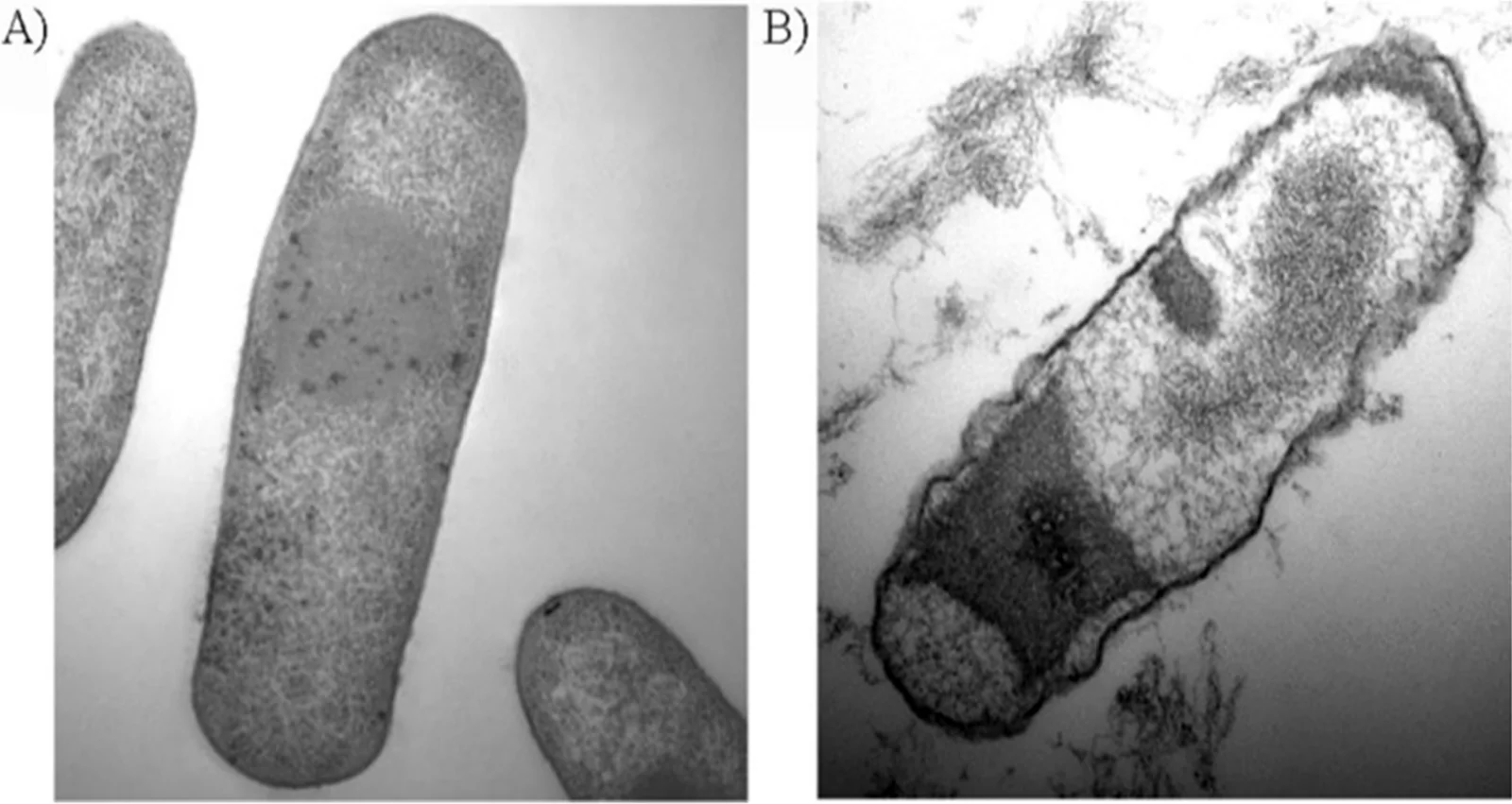
TEM images of *Escherichia coli* cells before any treatment **A** and after treatment with a suspension of L-menthol/oleic acid, 1:1. With the permission of Cao et al. (2020)

Electron microscopy serves as a powerful investigative tool in the exploration of the underlying mechanism of action associated with NADES as antimicrobial agents. The high resolution of scanning electron microscopy (SEM) provides researchers with valuable insight into the nature of interactions between NADES and microbes. A prominent application of SEM in these studies involves the close examination of their impact on the ultrastructure of microorganisms. Through the testing of NADES on the microorganism, followed by subsequent analysis using electron microscopy, researchers are able to report any observable alterations in cellular morphology or organelle structure, such as disrupting the integrity of the cell membrane, changing membrane permeability, or inducing a leakage of cellular contents. In this context, SEM plays an important role in elucidating the intracellular targets of antimicrobials by visualizing the internal structure of microorganisms. The SEM images in these experiments help determining the specific sites of action of NADES as antimicrobials. In addition, electron microscopy offers invaluable insights into the localization and distribution of these agents within microorganisms. Researchers can effectively employ electron-dense markers to label NADES, thereby facilitating the tracking of their uptake and subcellular localization (Durand et al. 2021). This information significantly aids in determining whether antimicrobial agents accumulate in specific organelles or interact with particular cellular components, thereby contributing to an enhanced understanding of their mechanism of action. Figure 5 illustrates the TEM images of *Escherichia coli* cells before and after treatment with the tested DES.

## Antibacterial activities of NADESs

### Antibacterial activity of NADESs against gram positive bacteria

Several NADESs were tested on gram-positive cocci, including *Staphylococcus aureus*, which can lead to inflammatory diseases such as skin infections, pneumonia, endocarditis, septic arthritis, osteomyelitis, and abscesses. It can also cause toxic shock syndrome (TSST-1), scalded skin syndrome (exfoliative toxin), and food poisoning (enterotoxin). *Methicillin-resistant Staphylococcus aureus* (*MRSA*), *Methicillin-resistant Staphylococcus epidermis* (*MRSE*), and *Enterococcus faecalis* are among the strains that have received great attention from the WHO due to their serious complications (Jubeh et al. 2020). *Streptococcus pneumoniae* was also used to evaluate the antibacterial activity of NADESs.

Among the gram-positive rod-shaped bacteria, spore-forming *Clostridium perfringens* (Wojeicchowski et al. 2021) and *Bacillus subtilis* (Mouffok et al. 2023) were tested. *Listeria monocytogenes*, a non-spore-forming gram-positive rod, was also included in the evaluation. *Listeria monocytogenes* can cause neonatal meningitis, meningitis in immunocompromised patients, gastroenteritis, and septicemia.

The best NADES that demonstrated large inhibition zones for *MRSA*, *MRSE*, *and Staphylococcus aureus* was menthol/lauric acid at a molar ratio of 4:1 (Oliveira et al. 2023). The inhibition zone sizes were 16.70 ± 0.47mm, 17.16 ± 0.62mm, and 14.17 ± 0.62mm, respectively, as measured by the disc diffusion method. The MIC for these bacteria, determined by the broth micro dilution assay using the same NADES, was 1250 µg/mL for *MRSA* and *MRSE*, and 625 µg/mL for *Staphylococcus aureus*. The results of the disc diffusion assay were compared to the positive control, gentamicin antibiotic, which showed inhibition zones of 19 mm for *MRSA*, 27.83 ± 0.62 mm for *MRSE*, and 36.33 ± 0.58 mm for *Staphylococcus aureus* (Oliveira et al. 2023).

It was also observed that capric acid/lauric acid at a molar ratio of 2:1 exhibited strong antimicrobial activity against *MRSA* and *MRSE*, with inhibition zone sizes of 16.50 ± 0.41 mm and 20.00 ± 0.82 mm, respectively. The inhibition zone for *Staphylococcus aureus* with the same NADES was 15.67 ± 0.58 mm. MIC values for these three strains were all equal to 625 µg/mL. Similar results were obtained for gentamicin as the positive control (Silva et al. 2019). These findings show the high potential of fatty acid-based NADES as antibacterial agents.

The largest inhibition zone for *Staphylococcus aureus* was achieved using choline chloride/oxalic acid at a molar ratio of 1:1, with an inhibition zone of 73 ± 3 mm (Radošević et al. 2018). Choline chloride/acetic acid at a molar ratio of 1:2 produced a strong inhibition zone of 50 ± 5 mm for spore-forming gram-positive bacteria *Bacillus subtilis* used (Mouffok et al. 2023). The MIC for *Bacillus subtilis* using the same NADES was 0.012 ± 0.002 µL/mL, as determined by the broth micro dilution method. *Clostridium perfringens* (Wojeicchowski et al. 2021), another spore-forming gram-positive bacteria associated with gas gangrene and food poisoning, showed an inhibition zone of 13–17 mm when treated with choline chloride/1,2-propanediol at a molar ratio of 1:2. Choline chloride/acetic acid at a molar ratio of 1:2 was found to be the best NADES for *Listeria monocytogenes* and *Enterococcus faecalis*, with inhibition zones of 40 ± 2 mm and 50 ± 5 mm, respectively (Mouffok et al. 2023).

Previous studies have shown that NADESs with strong inhibition zones often contain choline chloride as the hydrogen bond acceptor (HBA). This can be attributed to the interaction between the cholinium cation in NADES and the polysaccharide chains of peptidoglycan through hydrogen bonding or electrostatic forces, resulting in cell wall damage (Wen et al. 2015). Organic acids are primarily used as the hydrogen bond donor (HBD) in NADESs, and their ability to lower the pH below optimal values (pH = 6.5–7.5) for bacterial growth contributes to the inhibition of bacterial growth by organic acid-based NADESs (Zhao et al. 2015). Table 1 compares the antibacterial effects of the reported NADES.

## Antibacterial activity of NADESs against gram-negative bacteria

As mentioned earlier, the WHO prioritized gram-negative bacteria in their list due to the presence of lipopolysaccharides on their outer cell wall. Gram-negative bacteria are often more resistant to antibiotics compared to gram-positive bacteria. These bacteria are associated with serious and life-threatening infections such as bloodstream, surgical site, complicated urinary tract, and lung infections (Rangarajan and Venkataraman 2020). They also contribute to increased mortality and morbidity rates. Some of the most challenging bacteria to treat include *Escherichia coli*, *Klebsiella*, *Acinetobacter*, *Pseudomonas* species, *Proteus mirabilis*, *Salmonella typhimurium*, and *Serratia marcescens* (Rangarajan and Venkataraman 2020). These bacteria exhibit resistance to multiple drugs and show increasing resistance against commonly available antibiotics. Additionally, they have the ability to develop new resistance mechanisms and transfer genetic material, making other bacteria drug-resistant as well. Therefore, it is crucial to search for alternatives to antibiotics as their effectiveness diminishes over time.

**Fig. 6:**
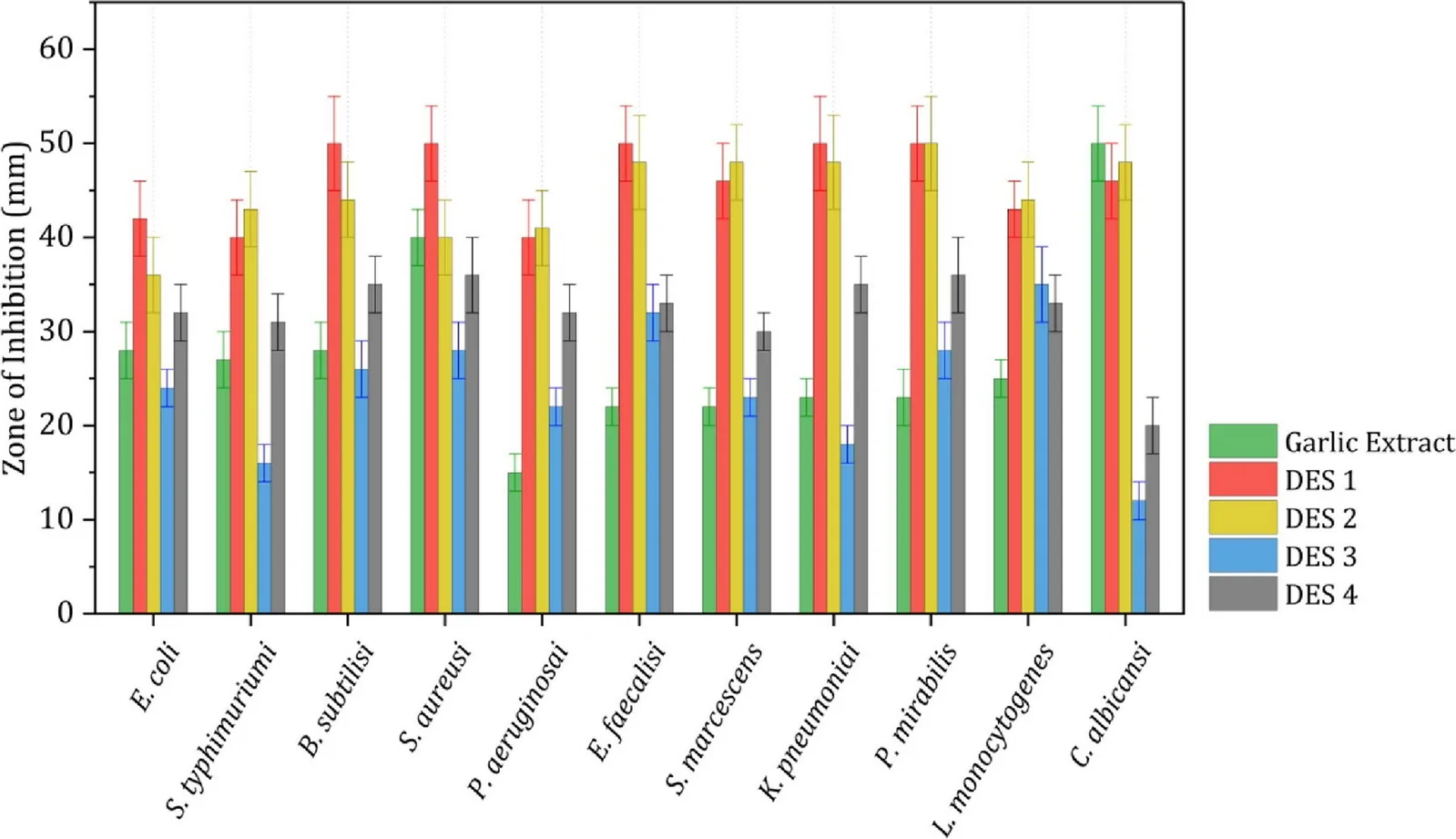
The antibacterial potential of garlic extract and the NADESs used with the permission of Mouffok et al. (2023)

Different NADESs were tested on a panel of gram-negative bacteria, and the inhibition zone was determined. According to the literature, citric-based NADESs demonstrated the highest toxicity towards gram-negative bacteria. Specifically, citric acid/fructose/glycerol at a molar ratio of 1:1:1 was tested on *Escherichia coli*, *Pseudomonas aeruginosa*, *Proteus mirabilis*, and *Salmonella typhimurium*, resulting in inhibition zones of 50 ± 4 mm, 51 ± 4 mm, 81 ± 2 mm, and 55 ± 1 mm, respectively. This was reported as the largest inhibition zone achieved for these bacteria using NADESs (Radošević et al. 2018). In the same study, choline chloride/oxalic acid at a molar ratio of 1:1 was tested on various gram-positive and gram-negative bacteria (Radošević et al. 2018). It was observed that gram-positive bacteria exhibited larger inhibition zones compared to gram-negative bacteria. This can be attributed to the additional outer lipopolysaccharide membrane present in gram-negative bacteria, making them less permeable than gram-positive bacteria. Consequently, choline chloride/oxalic acid had a lesser inhibitory effect on *Escherichia coli* (47 ± 2 mm), *Proteus mirabilis* (49 ± 1 mm), *Salmonella typhimurium* (45 ± 4 mm), and *Pseudomonas aerugino*sa (50 ± 2 mm) compared to *Staphylococcus aureus* (73 ± 3 mm) (Radošević et al. 2018). For *Klebsiella pneumoniae* and *Serratia marcescens*, the inhibition zones were 50 ± 5 mm and 45 ± 5 mm, respectively. These results were obtained using choline chloride/acetic acid at a molar ratio of 1:2. The MIC values for *Klebsiella pneumoniae* and *Serratia marcescens* were 0.10 ± 0.05 µL/mL and 0.001 ± 0.005 µL/mL, respectively. Figure 6 illustrates the antibacterial potential of garlic extract and the NADESs used.

It was also observed that these acid-based NADESs exhibited the strongest inhibition zones against bacteria compared to other NADESs (Mouffok et al. 2023). Therefore, it can be concluded that NADESs containing acids demonstrated higher antibacterial activity. Similar results and explanations apply to gram-positive bacteria as well (Zhao et al. 2015). Acids act as starting materials in NADESs and contribute to their antibacterial activities by creating an acidic environment, disrupting microbial membranes, and causing protein denaturation.

## Mechanism of action of NADES against bacteria and fungi

There are several factors that explain how these NADES induce toxicity against different microorganisms. Factors that affect NADES toxicity could be divided according to the structure of the microorganisms and the chemical composition of the starting materials of certain NADES (Marchel et al. 2022). In addition, the method of preparation of NADES could also affect their toxicity.

According to the structure of microorganisms, gram-negative bacteria have extra lipopolysaccharides on their outer cell wall. It was observed that the toxicity of NADES decreases in gram-negative compared to gram-positive bacteria, which do not have lipopolysaccharides (Radošević et al. 2018). For example, when choline chloride/oxalic acid in a molar ratio of 1:1 was tested on both gram-positive and negative bacteria, the inhibition zone was larger in gram-positive bacteria, supporting this assumption (Radošević et al. 2018). It was also observed that the toxicity of the same NADES was lower in fungi compared to bacteria. Fungi have a two-layered cell wall mainly composed of chitin and glucans, which makes it difficult for the NADES to penetrate their cell wall. This difference in fungal structure could explain why choline chloride/oxalic acid/glycerol and choline chloride/citric acid/glycerol were found to be toxic to bacteria but not to yeast *Candida albicans* (Jangir et al. 2020).

In addition to factors related to the chemical structure of the starting material of the tested NADES, it was mentioned previously that NADES consist of a hydrogen bond donor and a hydrogen bond acceptor, each of which affects NADES toxicity. Regarding the hydrogen bond donor, it was found that organic acid-based NADES showed strong antimicrobial activity. This can be explained by the fact that bacteria and fungi have their optimum pH for growth, which is 6.5–7.5 for bacteria (Marchel et al. 2022) and 5.0–9.0 for fungi (Nevarez et al. 2009). Since organic acid-based NADES have pH values below 3, this high acidity increases their toxicity towards microbial cells. The acidic pH denatures proteins located on the microorganism’s cell wall, negatively affecting cell activity. This negative effect of pH theory is supported by the study of De Morais et al. (2015), who observed that the pH values of organic acid-based NADESs were lower than 3, resulting in protein denaturation and decreased *Aliivibrio fischeri* cell activity.

Continuing with the hydrogen bond donor, it was also reported that carbohydrate-based NADES have a negative effect on microbial cells. The highly viscous nature of carbohydrate-containing NADESs increases their toxic effect. Additionally, these NADESs have high osmotic pressure properties, which lead to negative dehydration effects that rapidly remove water from the microbial cell, causing cell dehydration and lysis. Moreover, high concentrations of the tested NADES generate high osmotic pressure, resulting in microbial cell dehydration. For example, in the work of Redovniković’s group, high concentrations of choline chloride/ethylene glycol and choline chloride/glucose caused high osmotic pressure and decreased viability of baker’s yeast cells (Cvjetko Bubalo et al. 2015). Therefore, high concentrations of carbohydrate-based NADES would increase their toxicity.

**Fig. 7:**
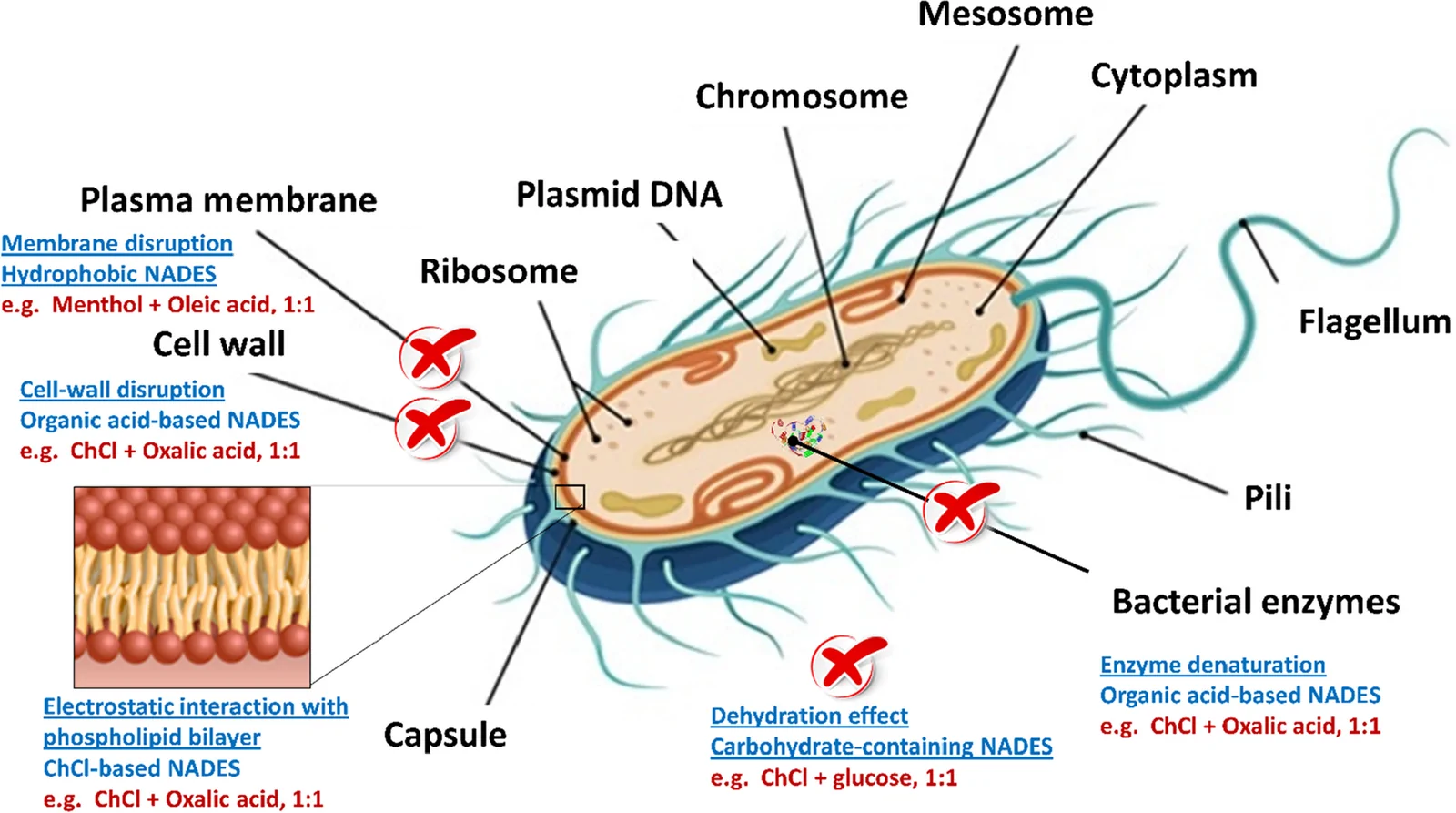
Different mechanisms of action of several NADESs

Regarding the hydrogen bond acceptor, it was observed that most of the HBA were choline chloride. This can be explained by the fact that choline chloride has a delocalized cation, and the higher toxicity of choline chloride-based NADESs is often attributed to the interaction of cholinium cation side chains and head groups with cellular membrane groups (Modica-Napolitano and Aprille 2001). It was also assumed that the accumulation of positively charged cations, such as cholinium, enhances electrostatic interactions with the negatively charged bilayer on the surface of cell membranes, leading to cell wall disruption (Wen et al. 2015). Finally according to method of preparation, it was found that heating and stirring method might result in impurities. The presence of impurities can change some of the mixture properties (e.g., by increasing their viscosities) and indirectly intensifying toxic effect of these NADESs (Florindo et al. 2014). Figure 7 summarizes different mechanisms of action of NADESs as antibacterial agents.

## Antifungal activity of NADESs

Fungal infections are one of the most significant public health concerns (Reddy et al. 2022). They can have severe complications in individuals, especially those with various disorders, including Covid-19. Fungal infections can lead to life-threatening mycoses and death when they occur as coinfections with Covid-19. The severity of fungal infections varies depending on the fungal species and the type of infection (Reddy et al. 2022). These infections range from superficial, cutaneous, subcutaneous, mucosal, to systemic infections. However, systemic infections are often diagnosed late, leading to increased mortality rates. One of the most important pathogens associated with serious fungal infections is *Candida* spp., which are part of the human microbiota and can cause opportunistic infections in healthy individuals. They can also cause life-threatening infections such as invasive candidiasis in immunocompromised people, including those infected with human immunodeficiency virus (HIV) or receiving chemotherapy for cancer, as well as patients on immunosuppressive drugs (Reddy et al. 2022).

In addition to opportunistic and systemic infections, fungal pathogens such as *Candida*, *Aspergillus*, *Fusarium*, *Mucorales*, and molds can cause healthcare-associated infections (HAIs) in patients with underlying diseases (Perlroth et al. 2007). Due to increasing resistance to current antifungal medications, alternative strategies must be considered for effective antifungal therapy (Reddy et al. 2022). One of these alternative strategies is the use of natural products (Reddy et al. 2022). Several NADES were tested against different panels of fungi to evaluate their antifungal activity. Among the tested NADES, choline chloride/oxalic acid at a molar ratio of 1:1 exhibited a strong inhibition zone against *Candida albicans* (Radošević et al. 2018). The inhibition zone was measured as 48 ± 3 mm using the disc diffusion method (Radošević et al. 2018). This result supports the finding that organic acid-based NADES have stronger antimicrobial activity compared to alcohol-, amine-, and sugar-based NADES (Zhao et al. 2015). This can be explained by the introduction of an additional hydroxyl group to organic acids, which increases their antimicrobial activity as the hydrogen bond donor of the NADES (Zhao et al. 2015).

## Other applications of NADES

In addition to the antimicrobial activity of NADES, they are also effective against biofilms, which represent a significant obstacle to the effective treatment of various infections in skin and soft tissues (Nystedt et al. 2023). NADES are also cytotoxic against tumor cells (Popović et al. 2023). Besides, it was reported that some NADES have a role in the extraction and purification of virus like particles (Marchel et al. 2020). Moreover they play an important role in antibacterial photodynamic therapy. These applications prove that NADES are promising alternatives to currently used materials not only as antimicrobials, but also in other fields of science.

## Conclusion

Natural deep eutectic solvents (NADES) are considered one of the most promising classes of alternative organic solvents. Their properties, including low flammability, non-toxicity, low volatility, low cost, biodegradability, and ease of preparation, make them widely used in diverse fields, ranging from pharmaceuticals to energy. The fact that NADES exhibit antimicrobial properties against a panel of microorganisms opens up possibilities for their use as alternatives to classical antibiotics. Their antimicrobial activity has been observed to be strong and efficient when based on organic acids. However, it has been noted that the toxicity of NADES varies according to the type of bacteria and cell line, which can be explained by the interaction of these systems with bacterial cell membranes. Currently, it is necessary to evaluate their biological activities, such as antimicrobial effects, cytotoxicity, and antioxidant properties, in order to identify the best solvent for large-scale industrial use. Rapidly acquiring knowledge about their toxicity is crucial for exploring their potential in various applications, particularly in biomedicine and the pharmaceutical industry.

## Data Availability

This manuscript has no associated data.

## Abbreviations

*ChCl*: Choline chloride
*CHX*: Chlorhexidine
*DES*: Deep eutectic solvents
*DNA*: Deoxyribonucleic acid
*EC50*: Effective concentration
*FTIR*: Fourier-transform infrared spectroscopy
*GCE*: Garlic clove extract
*HBA*: Hydrogen bond acceptor
*HBD*: Hydrogen bond donor
*ILs*: Ionic liquids
*KBr*: Potassium bromide
*MAS*: Microwave-assisted synthesis
*MIC*: Minimum inhibitory concentration
*MRSA*: Methicillin-resistant Staphylococcus aureus
*MRSE*: Methicillin-resistant Staphylococcus epidermis
*NADES*: Natural DES
*NR*: Not reported
*PCA*: Principle component analysis
*RNA*: Ribonucleic acid
*RTILs*: Room-temperature ionic liquids
*SEM*: Scanning electron microscopy
*TSST-1*: Toxic shock syndrome
*UAS*: Ultrasound-assisted synthesis
*VOCs*: Volatile oil compounds
*WHO*: World Health Organization
